# Update on the Phenotypic and Genotypic Spectrum of *KIF11*-Related Retinopathy

**DOI:** 10.3390/genes13040713

**Published:** 2022-04-18

**Authors:** You Wang, Zhaotian Zhang, Li Huang, Limei Sun, Songshan Li, Ting Zhang, Xiaoyan Ding

**Affiliations:** State Key Laboratory of Ophthalmology, Zhongshan Ophthalmic Center, Sun Yat-Sen University, Xianlie S Road, Guangzhou 510000, China; wangy998@mail2.sysu.edu.cn (Y.W.); zhangzhaotian@gzzoc.com (Z.Z.); huangli@gzzoc.com (L.H.); sunlimei@gzzoc.com (L.S.); lisongshan@gzzoc.com (S.L.); zhangting@gzzoc.com (T.Z.)

**Keywords:** familial exudative vitreoretinopathy, *KIF11*, chorioretinal dysplasia, increase and straightening of peripheral vessels

## Abstract

Background: This study aimed to report the frequency of *KIF11*-mutations in a large familial exudative vitreoretinopathy (FEVR) population, extend the clinical spectrum of *KIF1*1-associated retinopathy and compare *KIF11*-associated retinopathy to FEVR with mutations in other genes. Methods: Genetic data collected from 696 FEVR families were reviewed. The ocular phenotypes in patients with *KIF11* mutations were analyzed and compared with those of FEVR patients with mutations in other genes (*FZD4*, *TSPAN12*, *LRP5*, *NDP* and *JAG1*). Results: In a cohort of 696 FEVR families, disease-causing *KIF11* mutations were identified in 3.6% of families (25/696). Among 25 *KIF11 mutations*, 80% (20/25) carried variants of loss of function and 48% (12/25) of variants were de novo. The phenotypes were variable. Compared with FEVR with disease-causing mutations in other genes, chorioretinal dysplasia was observed in 44.2% (31/70) of eyes with *KIF11*-associated retinopathy and in only 1.3% (1/70) of eyes with FEVR with mutations in other genes (*p* < 0.01). Increase and straightening of peripheral vessels (ISPV) was observed in 17.1% (12/70) of eyes with *KIF11*-associated retinopathy, and in 50% (39/78) of eyes with FEVR with mutations in other genes (*p* < 0.01). Conclusions: The frequency of the *KIF11* mutation in FEVR was 3.6% in our database. The manifestation of *KIF11*-associated retinopathy was variable and different from the phenotype in FEVR caused by other genes. Chorioretinal dysplasia, instead of retinal folds, was the dominant phenotype in *KIF11*-associated retinopathy. ISPV was rare in *KIF11*-associated retinopathy. Moreover, our study revealed that most pathogenic *KIF11* mutations were de novo.

## 1. Introduction

The kinesin family member 11 (*KIF11*) gene is located on 10q23.33 and encodes a protein called kinesin family member 11 or EG5 [1]. Mutations in *KIF11* have been known to be a cause of an autosomal dominant inherited disease characterized by microcephaly with or without chorioretinopathy, lymphedema, or mental retardation (MCLMR: OMIM#152950, ORPHA#2526) [2,3]. In 2014, a *KIF11* gene defect was identified as one of the causes of familial exudative vitreous retinopathy [4], which is characterized by arrested retinal angiogenesis and its complications, including avascular areas, macular ectopia, retinal folds and retinal detachment [5,6]. So far, several genes have been identified in nearly half of the index cases with FEVR; these include *LRP5*, *FZD4*, *TSPAN12*, *NDP*, *CTNNA1*, *CTNNB1, KIF11* and *ZNF408* [7,8,9]. Unlike those caused by defects in other genes, the mechanism of *KIF11* in FEVR is different. Recently, it has been proposed that *KIF11*-associated retinopathy varies with FEVR, and several cases have been reported to verify this suggestion [10]. To our knowledge, no large case series of *KIF11*-related retinopathy have been reported.

In the study, to fill this gap and identify the possible differences between FEVR with mutations in other genes, we recruited a large cohort of 35 patients with *KIF11* variants from 25 families and compared the ocular phenotypes with those of 39 FEVR patients with mutations in other genes.

## 2. Materials and Methods

### 2.1. Patient Cohort

This study was carried out following the tenets of the Declaration of Helsinki and was approved by the Medical Ethics Committee of the Zhongshan Ophthalmic Centre (ZOC) at Sun Yat-sen University. Informed consent for the use of clinical data and to provide blood samples for research purposes was obtained from all the patients; for patients under 18 years old, informed consent was obtained from their parents.

A retrospective review was performed on 696 unrelated FEVR families between May 2010 and May 2021 at the ZOC. Targeted next-generation sequencing or whole exome sequencing was performed in all probands. For comparison of *KIF11*-associated retinopathy with FEVR associated with other genes, 39 genetically confirmed individuals from 27 families with FEVR caused by other genes (*FZD4*, *TSPAN12*, *LRP5*, *NDP* and *JAG1*) between December 2018 and December 2020 were enrolled for comparison.

### 2.2. Clinical Data

A full medical and family history was obtained for all patients, and the manifestation of syndromic conditions was recorded. We reviewed the clinical data of all patients and their family members, including visual acuity and intraocular pressure measurements. A slit lamp examination (cornea, iris, and lens), RetCam III image (130° lens, Clarity Medical Systems, Inc. Pleasanton, CA, USA), fundus fluorescein angiography (FFA), scanning laser ophthalmoscopy (SLO, California FA, Optos, Dunfermline, UK) examination, swept-source optical coherence tomography (SS-OCT, VG200, SVision Research, Shanghai, China) and electroretinography (ERG) were also performed on the available patients; ten family members also underwent FFA and SLO. All images were read by two experienced ophthalmologists (D.X. and W.Y). Chorioretinal dysplasia is identified as the segmental loss of choroid, retinal pigmental epithelium and the outer nuclear layers, and is characterized by scleral hyper-reflectivity and sharply demarcated zones. Retinal folds are identified as a series of lines that appear as yellowish or dark bands and are involved in the posterior pole, including peripheral neovascularization, peripheral retinal traction with temporal dragging and macular dragging [11]. Paving-stone degeneration is defined as a form characterized by small, discrete and rounded areas of depigmentation and retinal thinning [12]. Retinal degeneration was identified when paving-stone degeneration or lattice degeneration were examined. The increase and straightening of peripheral vessels (ISPV) are identified as a change in number or form in the vascular branch [13]. The end-stage of the disease is defined as retinopathy with complications that cause severe impairment of visual acuity (e.g., total retinal detachment, neovascular glaucoma, corneal degeneration or opacification, vitreous hemorrhage and anterior chamber disappearance) [14].

### 2.3. Mutational Screening

A total of 0.5 mL of peripheral venous blood was extracted following the manufacturer’s recommendations from all the probands and available family members for DNA analysis. Targeted next-generation sequencing (TGS) was performed from 1 January 2015, to 31 December 2017, which was based on a panel of 126 targeted genes, including *NDP, FZD4, LRP5, TSPAN12* and *KIF11* [15]. Whole exome sequencing was performed starting on 1 January 2018, the process was described in one previous study [16]. To identify the variants, 11 genes associated with FEVR, including *NDP*, *FZD4*, *LRP5*, *TSPAN12*, *ATOH7*, *ZNF408*, *KIF11*, *RCBTB1*, *JAG1*, *CTNNB1* and *CTNNA1*, were analyzed. Furthermore, Sanger sequencing was further used to verify *KIF11* gene variants and was performed on the family members, including both parents of 24 probands, for segregation analysis. The Human Gene Mutation Database (HGMD) and the Genome Aggregation Database (gnomAD) were used in the bioinformatic analysis of the variants [17,18]. Variants with minor allele frequencies (MAF) > 0.01 were filtered out. A panel consisting of SIFT, Polyphen2, Mutation Taster, Protein Variation Effect Analyzer (PROVEN) and Combined Annotation Dependent Depletion (CADD) was used to identify variants with predicted pathogenicity. Annotation of the splicing site was verified using VariantValitor, and the predicted effect on splicing was assessed with Human Splicing Finder, BDGP Splice Site Prediction and NetGene2. The pathogenicity of the mutation was predicted by comparing the multiple in silico predictions and the allelic frequencies, as well as the available segregation analysis. Otherwise, variants were classified as variants of unknown significance (VUS). The classification of pathogenic variants was defined on the basis of the guidelines of the American College of Medical Genetics [19]. Patients with benign variants were excluded from this study.

### 2.4. Statistical Analysis

All results were analyzed using SPSS 22.0 for Windows (IBM Corporation, Armonk, NY, USA). Continuous variables were presented as mean ± standard deviation ($\bar{X}$ ± S) and analyzed with the Student’s *t*-test. The chi-squared or Fisher’s exact test was used for categorical data. A *p*-value less than 0.05 was considered to be statistically significant.

## 3. Results

### 3.1. Clinical Features of KIF11-Related Retinopathy

**Figure 1:**
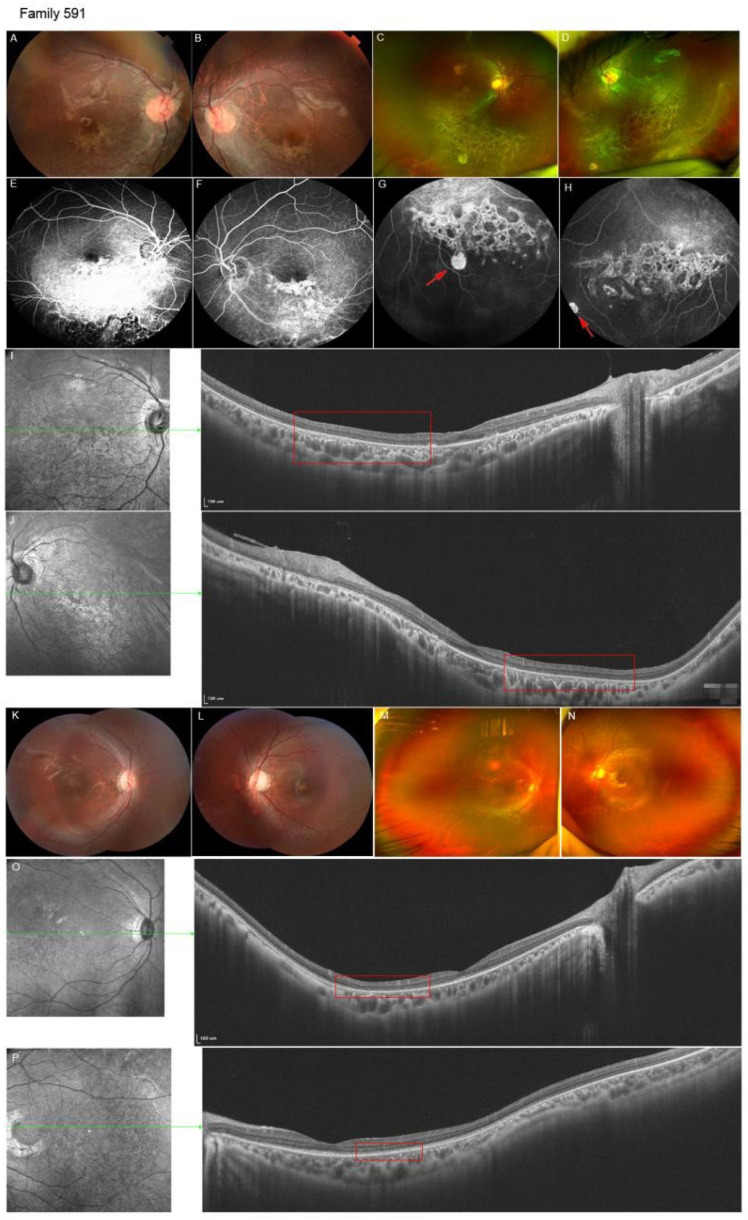
The c.1915dupA was detected in an eight-year-old boy and his younger brother. Ocular materials of proband (**A**–**J**): The fundus photograph showed symmetric inferior and para-optic chorioretinal dysplasia in both eyes, where the macula was involved (**A**,**B**). SLO showed isolated chorioretinal dysplasia unrelated to folds and paving-stone degeneration (red arrow) in both eyes (**C**,**D**). FFA revealed symmetric hyperfluorescence corresponding to lesions in both eyes (**E**–**H**). SS-OCT showed focus loss of the photoreceptor layers (red frame) in both eyes, the outer plexiform layer and outer nuclear layer in the macula were affected (**I**,**J**). Ocular material of his brother (**K**–**P**): Fundus imaging showed bilateral para-optic chorioretinal dysplasia and a pale optic disc in the left eye (**K**,**L**). SLO showed normal peripheral retina (**M**,**N**). SS-OCT showed a focus loss of photoreceptor layers (red frame) located in the temporal retina in both eyes (**O**,**P**).

**Figure 2:**
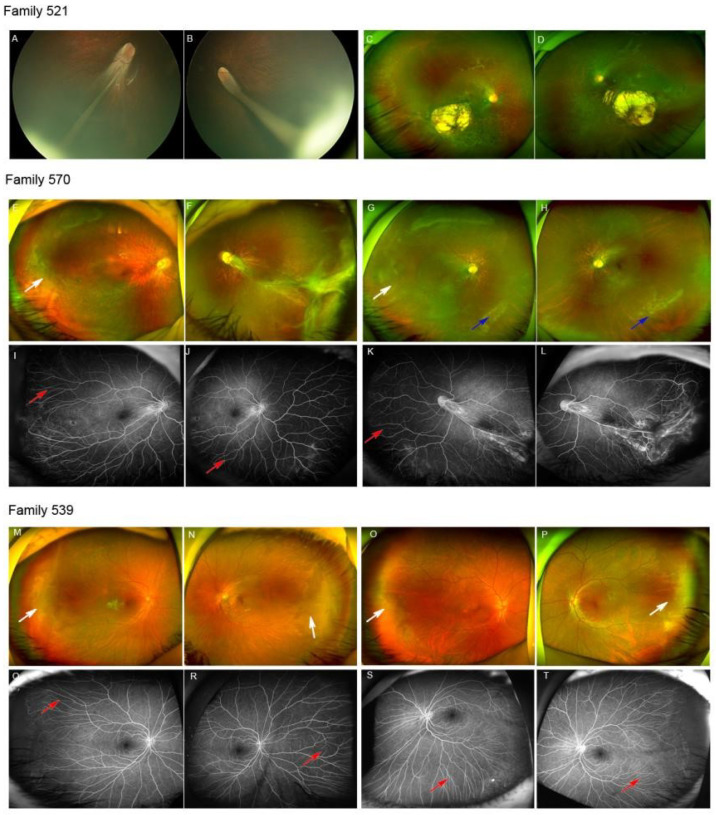
The c.2309_2310del was detected in a 5-month-old girl. The Retcam showed bilateral retinal folds in both eyes (**A**,**B**), while the SLO of her father showed symmetric chorioretinal dysplasia and exposed sclera located in the inferior and temporal retina (**C**,**D**). The c.139C>T was identified in a 5-year-old boy. The SLO demonstrated temporal mid-peripheral vitreoretinal interface abnormality (TEMPVIA) in the right eye (white arrow) and retinal folds in the left eye (**E**,**F**). The SLO of his mother showed TEMPVIA (white arrow) and lattice degeneration (blue arrow) in the peripheral retina (**G**,**H**). The FFA of the proband showed ISPVs in the temporal retina, nasal retina and inferior retina (**I**–**L**). The c.3073A>G was identified in a 5-year-old boy and his father. The SLO showed bilateral TEMPVIA (white arrow) in the temporal retina (**M**,**N**). His father’s SLO similarly showed mild changes (**O**,**P**). The FFA of the proband revealed ISPVs (red arrow) in the temporal retina, nasal retina and inferior retina (**Q**–**T**).

*KIF11* mutations were detected in 3.6% (25/696) of the families. Nine of these were reported in our previous study [18]. The clinical manifestations of all 25 probands and their 10 family members were analyzed in the current study (Table 1). The phenotypes were highly variable, including the peripheral avascular zone, ISPV, temporal mid-peripheral vitreoretinal interface abnormality (TEMPVIA), lattice degeneration, paving-stone degeneration, secondary choroidal coloboma, chorioretinal dysplasia, retinal folds, retinal detachment and loss of photoreceptor detected by SS-OCT. In a total of 35 subjects with *KIF11* variants, chorioretinal dysplasia was found in 44.2% (31/70) of eyes and was the leading phenotype of *KIF11*-associated retinopathy, followed by retinal folds (34.3%, 24/70) and retinal degeneration (12.9%, 9/70). End-stage FEVR, total tractional retinal detachment, was detected in 20% (14/70) of eyes. TEMPVIA was detected in six eyes from one proband and three family members. However, ISPV was seen in 17.1% (12/70) of eyes. Thus, we suggest that chorioretinal dysplasia is the most common feature of *KIF11*-associated FEVR (Table 1). The representative cases are shown in Figure 1 and Figure 2.

### 3.2. Ocular Comparison between Two Subgroups

The male-to-female ratios in Group 1 (35 patients with *KIF11* variants) and Group 2 (39 patients with other gene variants) were 26:9 and 28:11, respectively. The median ages of the probands at diagnosis were 2.42 ± 2.42 years old (range: three months to eight years old, *n* = 24) in Group 1 and 3.65 ± 3.70 years old (range: one month to 13 years old, *n* = 27) in Group 2. No significant difference was found in age or gender between the two groups. The detailed information is summarized in Table 2.

Of a total of 39 patients in Group 2, ISPV was the dominant phenotype and was found in 50% (39/78) of eyes, followed by retinal folds (38.5%, 30/78) and end-stage retinopathy (10.3%, 8/78). Retinal degeneration was seen in 16 eyes. The difference in the frequency of chorioretinal dysplasia and vessel changes between the two groups was statistically significant. There was no statistical difference in retinal folds or retinal degeneration between the two groups. The details are provided in Table 3.

### 3.3. Genetic Findings

We detected 25 heterozygous variants in *KIF11* in 25 families. In total, there were 12 frameshifts and seven non-sense, four missense, one non-frame and one splicing mutation. Of the 25 total variants, nine *KIF11* variants, including five frameshifts, two missense, one nonsense and one splicing mutation, were reported in our previous study [20]. We report the remaining 16 variants in this study, including eight nucleotide substitutions (six of which resulted in premature stop codons, and two of which resulted in missense mutations), one in-frame, one non-frame and six frameshift mutations. Of the eight single nucleotide substitutions, c.2626C>T, c.1249G>T, c.116C>G, c.139C>T, c.2266C>T and c.2344G>T created a premature stop-gain that would lead to early truncation of encoded proteins, while the changes of c.3073A>G and c.2830C>T, which had been reported by Li and Ostergaard, respectively, were predicted to be pathogenic variants after several pathogenic analyses [2,19]. The non-frame mutation c.1288_1290del resulted in a loss of amino acids, and it is also predicted to be pathogenic even though the reading frame remains unchanged. Both in-frame mutations and frameshifts can cause significant changes in the protein structure and may be deleterious. Fourteen novel variants were detected; the detailed information is presented in Table 4. Of the 25 variants in this study, 12 (48%, 12/25) were de novo. Furthermore, 20 (76.9%, 20/25) were the result of loss of function (LOF) including frameshift (48%, 12/25), spicing defects (4%,1/25) and stop-gain (28%, 7/25).

### 3.4. Extraocular Phenotypic Characteristics

Microcephaly was detected in 76% (19/25) of the probands, and facial characteristics, including large prominent ears and a broad nose, were observed in 80% (20/25). Lymphedema was observed in 11 (56%, 14/25) probands. Mental retardation could not be evaluated in most children, as the probands’ ages were very young at their first appointment. Two eight-year-old probands were shown to be generally normal, except for mild learning difficulties.

## 4. Discussion

In 2012, mutations in the *KIF11* gene were first identified in an MCLMR cohort by Ostergaard [2]. In 2014, Robitaille found that a small group of cases of FEVR was caused by mutations in the *KIF11* gene [21]. To date, mutations of the *KIF11* gene have been identified in two different diseases: FEVR and MCLMR. FEVR is a congenital retinal vessel disease, and MCLMR is a multiple systems disorder [22,23,24]. Whether *KIF11*-related ocular anomalies belong to the clinical spectrum of FEVR-related retinopathy or whether they are part of a single MCLMR entity remains controversial. To gain further insight into *KIF11*-associated ocular anomalies, we presented a large FEVR cohort with *KIF11* mutations and, for the first time, compared it with a FEVR cohort unrelated to *KIF11*.

In this study, *KIF11* mutations were detected in 3.6% (25/696) families; the frequency is similar to our previous study and lower than that reported by Hu et al. The number of cases is an important factor in estimating frequency, and it is more accurate in a larger cohort. In line with their study, patients with *KIF11* mutations showed variable clinical manifestations ranging from asymptomatic to blindness.

### 4.1. Chorioretinal Dysplasia, Not Retinal Folds or Abnormal Vessels, Is the Dominant Ocular Phenotype in KIF11-Associated Retinopathy

Choroidopathy was found to be the dominant ocular feature in *KIF11*-related retinopathy in cases with MCLMR, which is commonly described as focal choroidal atrophy or dysplasia with variable phenotypes ranging from mild RPE damage to diffuse choroidal dysplasia [10]. The pathogenesis of choroidopathy is elusive; one explanation may be that loss of function in EG5 could hamper the normal development of RPE cells. To some extent, in vitro experiments support our hypothesis; immunostaining of *KIF11* in the murine retina showed that *KIF11* was located in the RPE photoreceptor layer and in the outer plexiform layers [25]; that inhibition of EG5 leads to faster but abnormal growth of neuron axons [26] and that defective neurogenesis may affect the normal function of RPE cells. Eventually, RPE damage may progress to a large area of RPE death and to subsequent chorioretinal dysplasia.

In our cohort studies, chorioretinal dysplasia was seen in 31 (44.2%) eyes in KIF11-associated retinopathy and in one (1.3%) eye in FEVR unrelated to *KIF11*, while retinal folds were noted in 24 (34.3%) eyes in *KIF11*-associated retinopathy and 30 (38.5%) eyes in FEVR unrelated to *KIF11*. ISPV was observed in 12 eyes in the *KIF11* group. This indicates that chorioretinal dysplasia is the critical difference between *KIF11*-associated retinopathy and FEVR unrelated to *KIF11*. Choroidopathy and retinal folds account for most of the *KIF11*-related ocular phenotypes, and chorioretinopathy accounted for the majority on further analysis. The current study is in line with previously published reports on MCLMR, which demonstrated that choroidopathy is the dominant phenotype in *KIF11*-associated retinopathy; this differs from FEVR unrelated to *KIF11*.

The prevalence of choroidopathy may be underestimated for several reasons. In severe cases in which the retinal structure is completely damaged and too blurred to be clearly observed, it is usually not possible to ascertain whether choroidopathy is accompanied by retinal folds. In addition, the early stages of choroidopathy may present as RPE damage or focal choroidal dysplasia in the far peripheral retina, which is difficult to identify directly with an ophthalmoscope. Even in the early stages of some cases, the fundus looks completely normal, and abnormal changes can only be detected with OCT or ERGs. Moreover, folds can be observed in many diseases, such as retinopathy of prematurity (ROP), Norrie disease, incontinentia pigmenti, congenital toxoplasmosis, and other syndromes [27,28]. Therefore, we suggest that choroidopathy or chorioretinal dysplasia, not retinal folds or abnormal vessels, is the dominant ocular phenotype in *KIF11*-associated retinopathy.

### 4.2. Rare Increase or Straightening of Peripheral Vessels Was Noted in KIF11-Associated Retinopathy

There was striking variability in the severity of *KIF*-associated retinopathy in this study. Retinal detachment and corneal degeneration at the end stage are not unique, but specific changes can easily be observed in the mild phenotype. In this study, ISPV was observed in 12 eyes with *KIF11*-associated retinopathy, which was noted in 39 eyes with FEVR unrelated to *KIF11*. We suggest that ISPV is an ocular feature that can help distinguish *KIF11*-associated retinopathy from FEVR caused by other variants, but it is only useful in patients whose retina could be observed. Li reported seven probands with advanced FEVR (stage4 or above) and suggested that FEVR had a phenotypic overlap with MCLMR. In fact, retinal detachment can be caused by many diseases; it is more meaningful to detect the difference in *KIF11*-associated retinopathy with FEVR in mild patients.

In our previous study in 2020, the genotype and phenotype of a small series with a small sample size of nine FEVR patients associated with *KIF11* mutations were reported [15]. It was noted that most of the causative mutations were frameshift and nearly half the mutations were de novo, which was further confirmed by this current study. Interestingly, some new phenotypic findings, including chorioretinal dysplasia and the absence of ISPV, were first noted in the current study.

Overall, *KIF11*-associated retinopathy has many features that are different from FEVR, which has been proven to be caused by an interruption of the Wnt/β-catenin signaling pathway, which is formed by FZD4, the coreceptor gene LRP5, the FZD4 ligand gene Norrin and a component of the Norrin-FZD4 complex gene TSPAN12 [5,14]. Thus far, the role of *KIF11* in retinal angiogenesis is unclear. *KIF11* has been regarded as a regulator of ciliogenesis in a previous study and was associated with the ciliary apparatus of retinal photoreceptor cells [29]. According to the localization of *KIF11* in the modified primary cilium of retinal photoreceptor cells, *KIF11*-mediated disease was speculated to represent syndromic ciliopathy [10]. The mutation of *KIF11* is more likely to damage RPE and retinal photoreceptor cells and leads to chorioretinal dysplasia and retinal degeneration, which is in line with our findings.

## 5. Conclusions

Based on the findings reported here, our study extends and clarifies the previous understanding of *KIF11*-related ocular anomalies: that chorioretinopathy is the most frequent ocular phenotype, that isolated chorioretinal dysplasia is the pivotal feature and that ISPV is rare in *KIF11*-associated retinopathy. Moreover, our study confirms that most pathogenic *KIF11* mutations are LOF and de novo. The results provide insight into the diversity of the mutational spectrum of *KIF11*.

## Figures and Tables

**Table 1:** The ocular manifestations of 35 subjects with *KIF11* associated FEVR.

Family ID	Gender	Age at Diagnosis	BCVA (logMAR) OD/OS	Ocular Findings		Other Findings	Source
				OD	OS		
24	M	1Y	NA	Retinal folds, chorioretinal dysplasia	Chorioretinal dysplasia	None	Previously reported
171	M	5M	NA	Retinal folds, chorioretinal dysplasia	End-stage	Microcephaly, Microphthalmia in the left eye	Previously reported
213	F	6M	NA	Retinal folds	Retinal folds	Microcephaly, lymphedema	Previously reported
213F	M	29Y	0/1	Retinal folds, chorioretinal dysplasia	Retinal folds, chorioretinal dysplasia	NA	-
227	F	6M	NA	Retinal folds	Retinal folds	None	Previously reported
242	F	5M	NA	Retinal folds, secondary cataract	Retinal folds	Microcephaly, Microphthalmia in the right eye, lymphedema	Previously reported
242F	M	25Y	0/0	Chorioretinal dysplasia	Chorioretinal dysplasia	NA	Previously reported
242S	M	8Y	1.0/0.7	Chorioretinal dysplasia	Chorioretinal dysplasia	NA	Previously reported
298	M	6Y	0.1/LP	Chorioretinal dysplasia, TEMPVIA, ISPVs	Rhegmatogenous retinal detachment	None	Previously reported
301	M	3Y	NA	Retinal folds	Retinal folds	Microcephaly, lymphedema	Previously reported
336	M	4M	NA	End-stage	End-stage	Microcephaly, lymphedema	Previously reported
417	M	4Y	NA	Retinal folds	Retinal folds	Microcephaly, lymphedema	Previously reported
463	M	1Y	NA	Retinal folds, chorioretinal dysplasia	End-stage	Microcephaly, lymphedema	-
463F	M	37Y	0.1/0.1	Chorioretinal dysplasia	Chorioretinal dysplasia	NA	-
481	M	2Y	NA	End-stage, secondary cataract	Retinal folds, secondary cataract	Microcephaly, lymphedema	-
492	M	3M	NA	Retinal folds, chorioretinal dysplasia	Retinal folds, chorioretinal dysplasia	NA	-
521	F	5M	NA	Retinal folds	Retinal folds	Microcephaly, lymphedema	-
521F	M	28Y	NA	Chorioretinal dysplasia, lattice degeneration	Chorioretinal dysplasia, lattice degeneration	NA	-
550	F	5M	NA	End-stage	End-stage	Microcephaly, lymphedema	-
577	F	4M	NA	Retinal folds	Retinal folds	Microcephaly, lymphedema	-
591	M	8Y	0.4/0.7	Chorioretinal dysplasia, ISPVs	Chorioretinal dysplasia, ISPVs	None	-
591B	M	7Y	0.8/0.4	Chorioretinal dysplasia	Chorioretinal dysplasia	NA	-
591F	M	35Y	NA	Chorioretinal dysplasia, ISPVs	Chorioretinal dysplasia	NA	-
557	M	8Y	NA	End-stage	Retinal folds, chorioretinal dysplasia	Microcephaly, lymphedema	-
673	M	3Y	NA	End-stage	Retinal folds; chorioretinal dysplasia	Microcephaly, lymphedema	-
673M	F	NA	NA	End-stage	Chorioretinal dysplasia, lattice degeneration	NA	-
680	M	3Y	NA	End-stage	End-stage	Microcephaly	-
684	M	3Y	NA	Chorioretinal dysplasia	End-stage	Microcephaly	-
688	M	2Y	NA	Chorioretinal dysplasia	Retinal folds, chorioretinal dysplasia	Microcephaly, lymphedema	-
564	M	5M	NA	End-stage	End-stage	Microcephaly	-
570	M	5Y	0.8/0.8	Ectopic macula, TEMPVIA, ISPVs, lattice degeneration	Retinal folds, ISPVs, lattice degeneration	None	-
570M	F	29Y	NA	TEMPVIA, ISPVs, tilted disc, posterior staphyloma, lattice degeneration	Tilted disc, posterior staphyloma, lattice degeneration	NA	-
539	M	4Y	NA	TEMPVIA, ISPVs, lattice degeneration	TEMPVIA, ISPVs, lattice degeneration	None	-
539F	M	NA	NA	TEMPVIA, ISPVs, lattice degeneration	TEMPVIA, ISPVs, lattice degeneration	None	-
653	F	NA	NA	Chorioretinal dysplasia, retinal folds	Chorioretinal dysplasia, retinal folds	Microcephaly, lymphedema	-

F: father/female; M: mother/male; B: brother; S: sister; Y: year; M: month; NA: none available; BCVA: best corrected visual acuity; OD: oculus dextrus; OS: oculus sinister; TEMPVIA, temporal mid-peripheral vitreoretinal interface abnormality; ISPV, increase or straightening of peripheral vessels. Previously reported [15].

**Table 2:** Demographic information of the two groups.

	*KIF11* Associated Retinopathy	FEVR Caused by Other Genes	*p*
Patients	35	39	
Probands	25 (71.4%)	27 (69.2%)	0.92
Family member	10 (28.6%)	12 (30.8%)	
Eyes	70	78	
Gender			
Male	26 (74.2%)	28 (71.7%)	0.91
Female	9 (25.7%)	11 (28.3%)	
Age(years)	12.40 ± 14.12 (*n* = 32)	14.30 ± 14.57	
Probands	2.42 ± 2.42 (*n* = 24)	3.65 ± 3.70	0.16
Family member	24.75 ± 11.32 (*n* = 8)	28.25 ± 13.03	0.77
Genes			
* KIF11*	35 (100%)	0	
* FZD4*	0	21 (53.8%)	
* TSPAN12*	0	9 (23.1%)	
* LRP5*	0	5 (12.8%)	
* NDP*	0	3 (7.7%)	
* JAG1*	0	1 (2.6%)	

**Table 3:** Comparison of *KIF11* associated retinopathy and FEVR caused by other genes (*FZD4, TSPAN12, LRP5, NDP* and *JAG1*).

Phenotypes (Eyes)	*KIF11* Associated Retinopathy (*n* = 70 Eyes)	FEVR Caused by Other Genes (*n* = 78 Eyes)	*p*
Chorioretinal dysplasia	31 (44.2%)	1 (1.3%)	<0.01
Retinal folds	24 (34.3%)	30 (38.5%)	0.61
Retinal degeneration	9 (12.9%)	16 (20.5%)	0.21
ISPVs	12 (17.1%)	39 (50%)	<0.01
End-stage	14 (20%)	8 (10.3%)	0.07

**Table 4:** Variants identified in *KIF11*.

Family Number	cDNA Change	Amino Acid Change	Heredity	Variant Impact	Allele Frequency	Mutation Taster	PROVEN	CADD	Variation Type	Source
463	c.339delT	p.F113Lfs * 23	Paternal	Frameshift	NA	DC	NA	NA	P, PVS1	Novel
481	c.2905_2909del	K969Rfs * 18	Paternal	Frameshift	NA	DC	NA	NA	P, PVS1	Novel
492	c.1288_1290del	p.430del	De novo	Nonframeshift	NA	DC	D	22.3	P, PS2	Novel
521	c.2309_2310del	p.K771Ifs * 4	Paternal	Frameshift	NA	DC	NA	NA	P, PVS1	Novel
539	c.3073A>G	p.R1025G	Paternal	Missense	0.000054	Polymorphism	D	23.1	P, PP5	Li, J.K. et al. [19]
550	c.2626C>T	p.Q876 *	De novo	Stop-gain	NA	DC	NA	36	P, PVS1	Novel
577	c.1382_1383del	p.L461Rfs * 11	De novo	Frameshift	NA	DC	NA	NA	P, PVS1	Novel
591	c.1915dupA	p.I639Nfs * 14	Paternal	Frameshift	NA	DC	NA	22.4	P, PVS1	Novel
557	c.1249G>T	p.E417 *	Maternal	Stop-gain	NA	DC	NA	NA	P, PVS1	Novel
564	c.116C>G	p.S39 *	De novo	Stop-gain	NA	DC	NA	NA	P, PVS1	Novel
570	c.139C>T	p.R47 *	Maternal	Stop-gain	NA	DC	NA	NA	P, PVS1	Novel
653	c.134del	p.L45Xfs * 91	De novo	Frameshift	NA	DC	NA	NA	P, PVS1	Novel
673	c.2830C>T	p.R944C	Maternal	Missense	NA	DC	D	32	P, PP5	Ostergaard, P. et al. [2]
680	c.2266C>T	p.Q756 *	De novo	Stop-gain	NA	DC	NA	43	P, PVS1	Novel
684	c.2344G>T	p.E782 *	De novo	Stop-gain	NA	DC	NA	36	P, PVS1	Novel
688	c.1429delA	p.E478Kfs * 61	De novo	Frameshift	NA	DC	NA	NA	P, PVS1	Novel

Nine variants reported in our previous study are not shown here. * indicates the appearance of termination codon. NA: none available; D: damaging or deleterious; DC: disease causing; P: pathogenic; PVS: very strong evidence of pathogenicity; PS: strong evidence of pathogenicity; PM: moderate evidence of pathogenicity; PP: supporting evidence of pathogenicity.

## Data Availability

All raw data used during the study are available from the corresponding author by request.
